# The association between aortic stenosis and the risk of consecutive eyelid inflammatory diseases

**DOI:** 10.7150/ijms.108261

**Published:** 2025-02-18

**Authors:** Shu-Ling Peng, Tung-Lin Tsui, Ke-Hsin Ting, Yasser Nassef, Chia-Yi Lee, Jing-Yang Huang, Chao-Bin Yeh, Chia-Jui Weng, Shun-Fa Yang

**Affiliations:** 1 Institute of Medicine, Chung Shan Medical University, Taichung, Taiwan.; 2 Division of Cardiology, Department of Internal Medicine, Camillian Saint Mary's Hospital Luodong, Luodong, Yilan, Taiwan.; 3 Division of Cardiology, Department of Internal Medicine, Changhua Christian Hospital, Yunlin Branch, Yunlin, Taiwan.; 4 Department of Nursing, Hungkuang University, Taichung, Taiwan.; 5 Department of Post-Baccalaureate Medicine, College of Medicine, National Chung Hsing University, Taichung, Taiwan.; 6 Nobel Eye Institute, Taipei, Taiwan.; 7 Department of Medical Research, Chung Shan Medical University Hospital, Taichung, Taiwan.; 8 Department of Emergency Medicine, School of Medicine, Chung Shan Medical University, Taichung, Taiwan.; 9 Department of Emergency Medicine, Chung Shan Medical University Hospital, Taichung, Taiwan.; 10 Department of Food and Beverage Services, Tainan University of Technology, Tainan, Taiwan.

**Keywords:** aortic stenosis, blepharitis, hordeolum, epidemiology, TriNetX database

## Abstract

Aortic stenosis (AS) is a fatal cardiovascular disease characterized by an inflammatory response. Blepharitis and hordeolum are two eyelid conditions that also involve ocular inflammation. The aim of this study is to assess the association between AS and the subsequent development of eyelid inflammatory diseases. This retrospective cohort study included individuals diagnosed with AS, who were matched to a control group of non-AS individuals from the TriNetX database. A total of 431,027 individuals were assigned to both the AS and non-AS groups. The primary outcomes of this study were the incidence rates of eyelid inflammatory diseases, including blepharitis and hordeolum. Cox proportional hazards regression was used for statistical analysis. After the follow-up period, 4,464 cases of blepharitis were recorded in the AS group, compared to 3,139 in the non-AS group. Additionally, there were 2,184 cases of hordeolum in the AS group, compared to 1,724 in the non-AS group. Cox proportional hazards regression analysis revealed that the AS group had a significantly higher risk of developing blepharitis (P < 0.001) and hordeolum (P < 0.001) compared to the non-AS group. The AS group also showed significantly higher cumulative probabilities of both blepharitis and hordeolum than the non-AS group (both P < 0.001). In subgroup analyses, AS patients exhibited a higher risk of developing blepharitis than the non-AS population, except for the Asian population and individuals aged 20-45 years. Similarly, the AS group showed a higher risk of developing hordeolum than the non-AS group, with exceptions in the African and Asian populations and individuals aged 20-45 years. In conclusion, the presence of AS is associated with a higher risk of developing subsequent eyelid inflammatory diseases.

## Introduction

Aortic stenosis (AS) is a significant cardiovascular disease characterized by fibrosis, calcification, and thickening of the aortic valve structure 1, 2. In Western countries, the prevalence of AS is estimated to be 5 percent in individuals over the age of 65 3. Medical management is typically used for mild cases of AS, while surgery, such as aortic valve replacement, is recommended for more severe cases 4, 5. Although the survival rate following aortic valve replacement is generally favorable 6, 7, postoperative complications related to AS can lead to mortality 3.

The association between AS and several other diseases has been well-documented 2. For instance, diabetes mellitus is frequently observed in individuals with AS 8. Additionally, hypertension is closely linked to the development of AS, and the coexistence of hypertension can impact both the symptoms and severity of AS 9. In terms of serum lipids, hypercholesterolemia can damage the cardiovascular system, and dyslipidemia has been shown to influence the formation and progression of AS 10. Beyond these metabolic conditions, elevated inflammatory responses and cytokine levels have also been observed during the development of AS 11.

Blepharitis is an eyelid inflammatory disease caused by dysfunction of the meibomian glands, which can lead to ocular irritation and subsequently dry eye disorder 12, 13. Hordeolum, another acute eyelid disorder, is characterized by eyelid swelling, microbial infection, and an inflammatory response 14, 15. However, there is limited research examining the association between AS and subsequent eyelid disorders. Since AS is commonly associated with systemic inflammation 16, the inflammatory response in AS may extend beyond the cardiovascular system, potentially affecting other organs, including the eyes. As a result, the presence of AS could influence the risk of developing eyelid inflammatory diseases such as blepharitis or hordeolum, necessitating further investigation. Additionally, shared risk factors, along with the effects of AS on blood flow and immune responses, may contribute to the development of eyelid inflammation, highlighting the importance of exploring this potential relationship. Thus, the purpose of this study is to evaluate the potential association between AS and subsequent eyelid inflammatory diseases, including blepharitis and hordeolum. Subgroup analyses based on demographic and laboratory data were also conducted.

## Materials and Methods

### Data source

TriNetX is a global federated health research network giving access to many electronic medical records (diagnoses, medications, laboratory values, procedures, genomic information) across prominent healthcare organizations. This research was generated using the US Collaborative Network, which involves 67 healthcare institutes. The medical data available in the TriNetX database involve the International Classification of Diseases, Tenth Revision, Clinical Modification (ICD-10-CM) codes, sex, age, occupation, place of residence, educational level, socioeconomic condition, length of hospitalization if existed, laboratory examination codes, image exam codes, the surgery codes, the procedure codes, and the Anatomical Therapeutic Chemical (ATC) codes for medications. This study was approved by the Institute Review Board of Chung Shan Medical University Hospital (project code: CS2-23180).

### Individual selection

**Figure 1:**
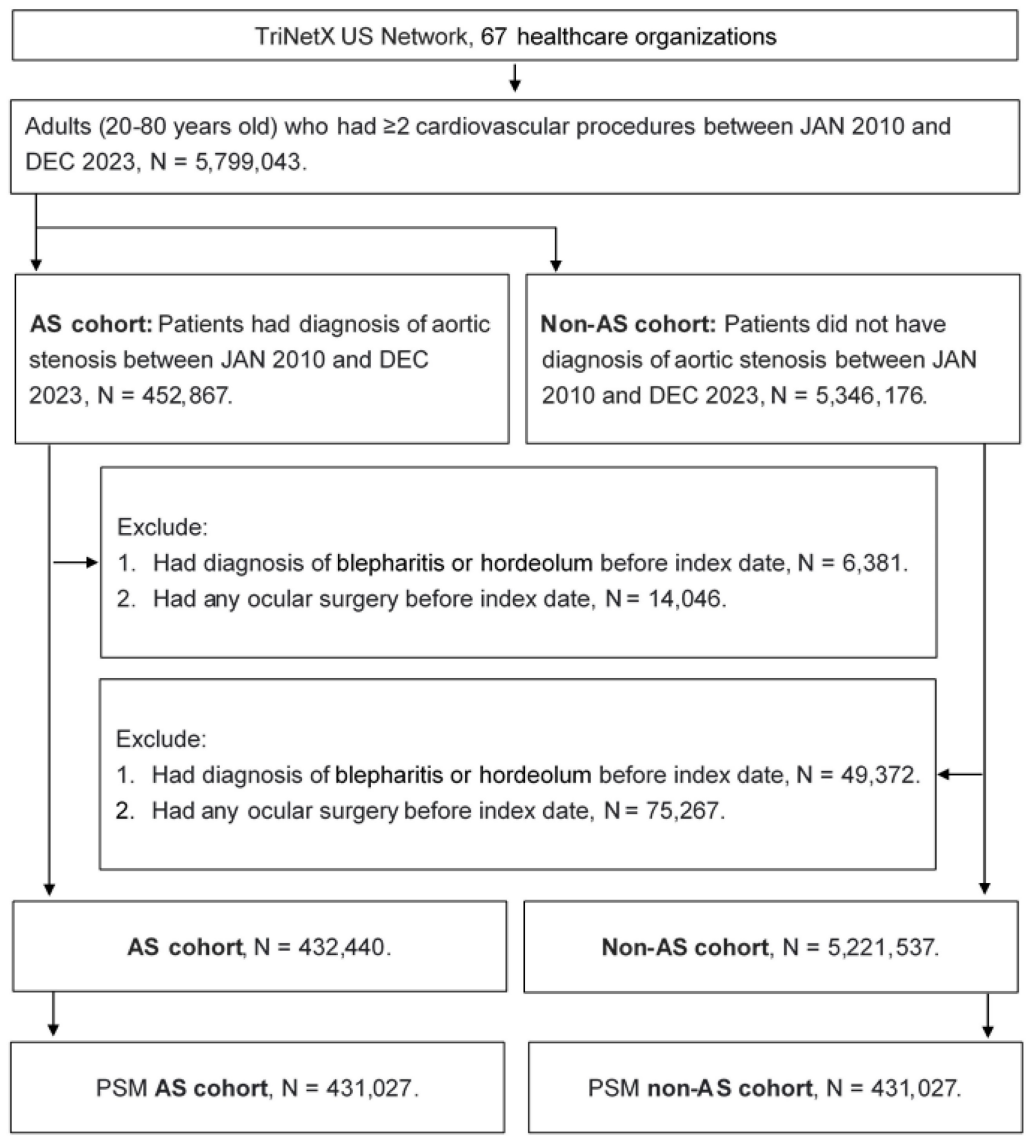
The flowchart of participant selection. AS: aortic stenosis, N: number, PSM: propensity score matching.

A cohort study was conducted, and individuals with the following characteristics were selected as AS cases: (1) a diagnosis of AS based on the relevant ICD-10 CM codes, (2) the receipt of an echocardiogram, electrocardiogram, chest X-ray, cardiac catheterization, or cardiac computed tomography prior to the AS diagnosis, and (3) the AS diagnosis was confirmed by a cardiologist. The index date in this study was defined as 6 months after the AS diagnosis. Exclusion criteria were applied to enhance the homogeneity of our study population: (1) individuals aged under 20 or over 80 years, (2) those who had undergone ocular surgery prior to the index date, and (3) those who experienced the outcome (described in the following section) before the index date. For the comparison, each individual with AS was matched to an individual without AS using the propensity score matching (PSM) process. The PSM process incorporates demographic information, systemic diseases, and medication use into a specific score and matches individuals based on these scores. After the process, a total of 431,027 and 431,027 individuals were put into the AS and non-AS groups, respectively. The flowchart of selection is revealed in Figure 1.

### Main outcome

The main outcomes of this study are the development of blepharitis and hordeolum. The blepharitis development was set as the subsequent conditions: (1) the receipt of blepharitis diagnosis by the related ICD-10 CM codes, (2) the arrangement of slit-lamp biomicroscope exam at the same day of blepharitis diagnosis by the procedure code, and (3) the prescription of antibiotic eyedrop or ointment by the ATC codes. In addition, the hordeolum development was set as the subsequent conditions: (1) the receipt of the hordeolum diagnosis by the related ICD-10 CM codes, (2) the arrangement of slit-lamp biomicroscope exam by the procedure codes, and (3) the application of topical antibiotic eyedrop or ointment by the ATC codes. Only the blepharitis and hordeolum episodes that developed after the index date were regarded as the outcome achievements in this study. All the individuals were followed until the outcome development, individual withdraw from all the available health insurance project or the end of TriNetX database which indicates the December, 31, 2023.

### Confounder adjustment

To better verify the association between AS and later eyelid inflammatory diseases, the effect of these confounders were adjusted in the multivariable analysis: age, urbanization, sex, income level, hypertension, hyperlipidemia, diabetes, ischemic heart disease, cerebrovascular disease, chronic kidney disease, chronic lower respiratory diseases, peripheral vascular disease, estimated glomerular filtration rate (eGFR), LDL, HDL, and Troponin I. The presence of above covariates is based on the related demographic, ICD-10 CM and laboratory codes. To confirm the durations of systemic morbidities are long enough to influence the possibility of eyelid inflammatory diseases, only the morbidities that persisted for longer than two years were chosen into the statistical analysis of this study.

### Statistical analysis

The SAS version 9.4 (SAS Institute Inc, Cary, NC, USA) was exploited in the statistical analyses in this study. The descriptive analysis was exploited to show the basic features of the AS group and the non-AS group, and the standard mean difference (SMD) was exploited to evaluate the difference of each index between the two groups. A SMD more than 0.1 was defied as significant difference in this study. Then the Cox proportional hazard regression was exploited to compare the incidences of blepharitis and hordeolum between the AS and non-AS groups, and the adjusted hazard ratio (aHR) with 95% confidence interval (CI) for the incidences of eyelid inflammatory diseases were produced. The Kaplan-Meier curve was pictured and the cumulative incidences of eyelid inflammatory diseases episodes between the two groups were calculated by the log-rank test. In the subgroup analysis, the patients were categorized into different subgroup based on age, sex, HDL, LDL and eGFR. Then the Cox proportional hazard regression was exploited again to analyze the risk of eyelid inflammatory diseases in different subgroups. Statistical significance was set as P < 0.05 and the P value beneath 0.001 was described as P < 0.001 in this study.

## Results

The initial features of the study population is illustrated in the Table 1. The mean age was 65.1±11.7 and 65.6±11.3 years old in the AS and non-AS groups, respectively. The difference of mean age between groups did not reach significant difference. The distributions of sex and urbanization were also similar between the two groups (both SMD < 0.1). About the systemic conditions, the rate of systemic diseases and laboratory data did not achieve significant difference between the AS and non-AS groups (all SMD < 0.1) (Table 1).

**Figure 2:**
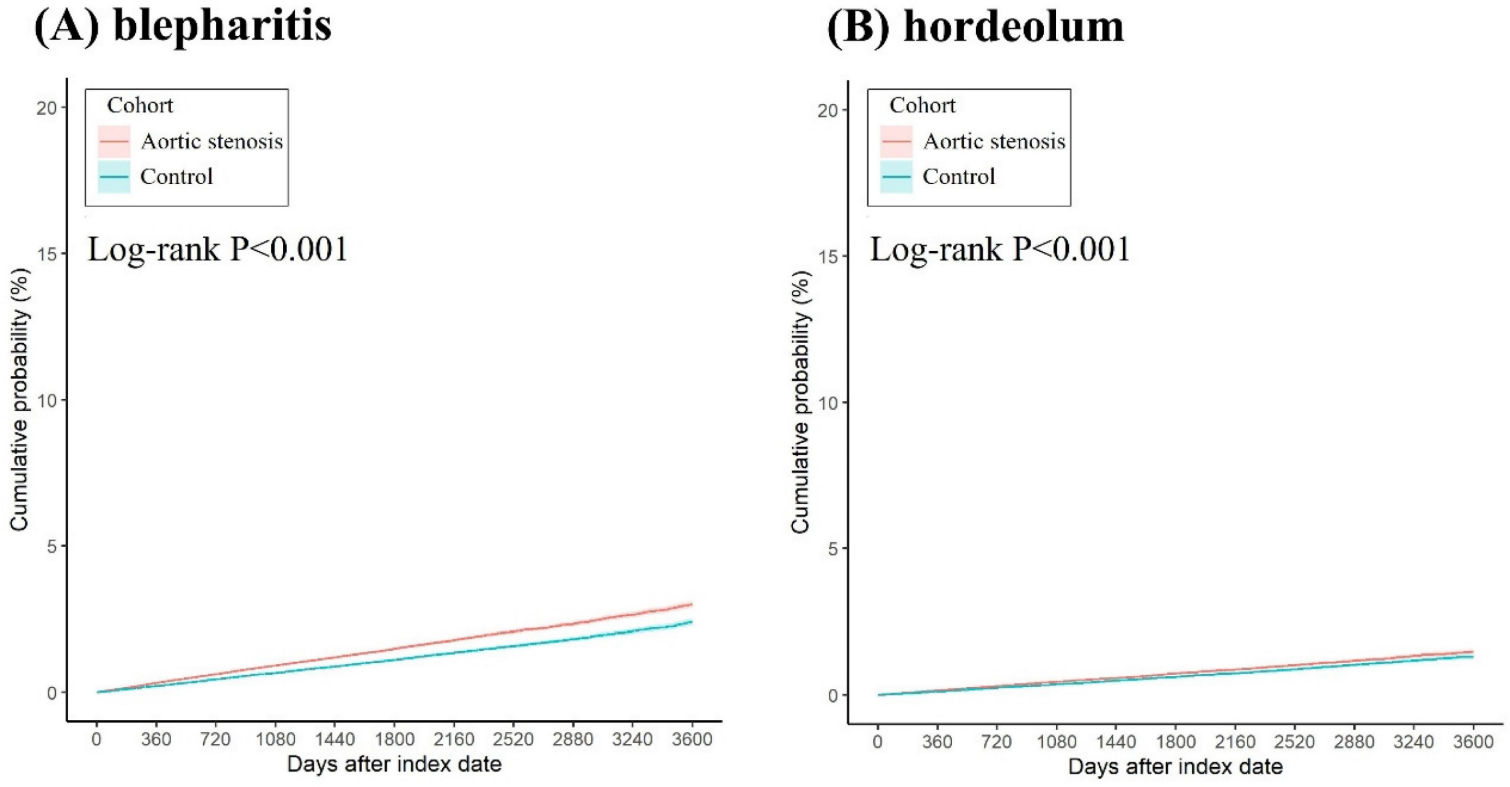
The Kaplan-Meier curve and cumulative incidence of (A) blepharitis events, and (B) hordeolum events between groups.

After the follow up interval, a total of 6,165 and 4,546 eyelid inflammatory diseases were found in the AS and non-AS group. There were 4,464 and 3,139 blepharitis events in the AS and non-AS groups, respectively. In addition, there was 2,184 and 1,724 hordeolum events in the AS and non-AS groups, respectively. According to the Cox proportional hazard regression, the AS group revealed a significantly higher risk of blepharitis development (aHR: 1.331, 95% CI: 1.271-1.393, P < 0.001) than the non-AS groups (Table 2). Also, the risk of hordeolum was significantly higher in the AS group (aHR: 1.183, 95% CI: 1.111-1.260, P < 0.001) than the non-AS group (Table 2). The cumulative probabilities of blepharitis and hordeolum between groups are displayed in Figures 2A-2B. The AS group revealed a significantly higher cumulative probabilities of blepharitis and hordeolum than the non-AS group (all P < 0.001) (Figures 2A-2B).

**Figure 3:**
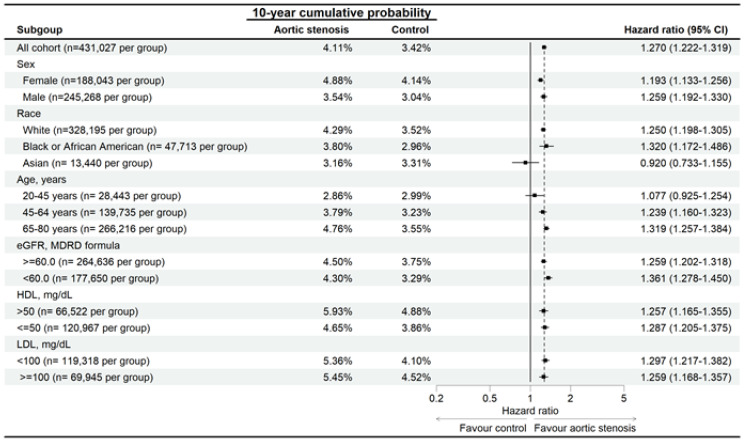
Risk of eyelid inflammatory diseases in patients with aortic stenosis stratified by age, sex, race, HDL, and LDL levels. N: number

**Figure 4:**
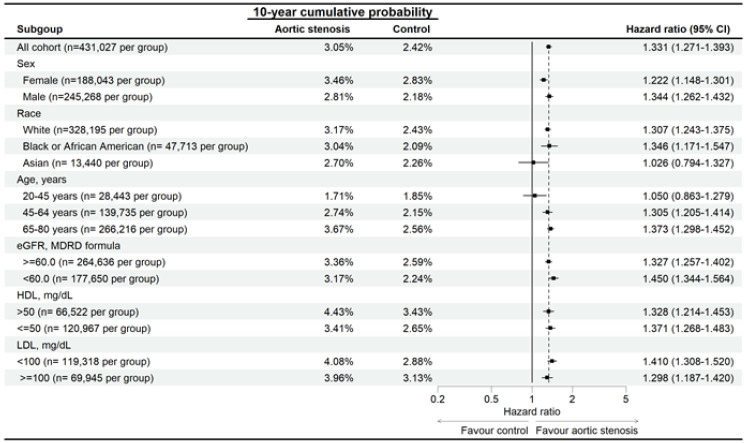
Risk of blepharitis in patients with aortic stenosis stratified by age, sex, race, HDL, and LDL levels. N: number

**Figure 5:**
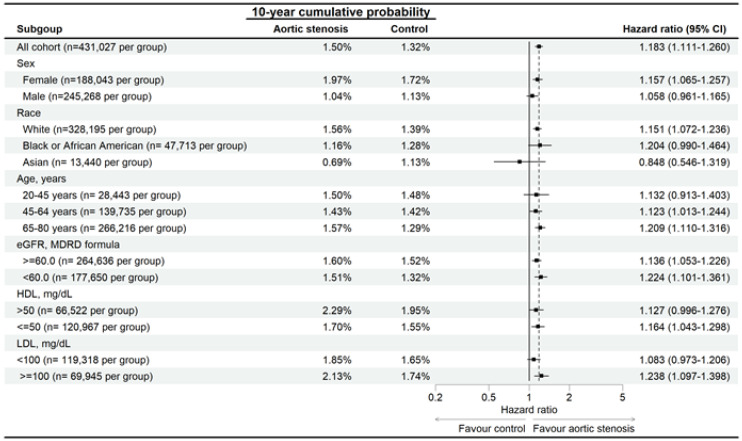
Risk of hordeolum in patients with aortic stenosis stratified by age, sex, race, HDL, and LDL levels. N: number

In the subgroup analysis, the AS patients with different characteristics demonstrated higher risk of developing eyelid inflammatory diseases than the non-AS population with same characteristics except the Asian population and those aged from 20-45 years (Figure 3). In addition, the AS patients also demonstrated higher risk of developing blepharitis than the non-AS population except for the Asian population and those aged from 20-45 years (Figure 4). On the other side, the AS subgroups revealed higher risk of hordeolum than the non-AS subgroup except for the African population, Asian population and those aged from 20-45 years (Figure 5).

## Discussion

In this study, the AS population demonstrated a significantly higher risk of blepharitis and hordeolum developments compared to the non-AS group after considering multiple demographic and disease factors. Moreover, the probability of developing eyelid inflammatory diseases in the AS groups was positively associated with disease interval. On the other hand, the correlation between AS and following eyelid inflammatory diseases are prominent in different populations except for the Asian population and individuals aged from 20-45 years.

The AS can associate with multiple diseases throughout the human body 17. The metabolic syndromes which referred to hypertension, glucose intolerance, and the dyslipidemia presented positive correlation to the existence of AS 18. Both the hypertension and hyperlipidemia would develop more common in the individuals with AS 9, 10. Moreover, the incidence of diabetes would elevate in the individuals diagnosed with AS, and the diabetes individuals with AS illustrated an advanced aortic valve calcification than the non-diabetes people 8. In addition to the metabolic syndromes, the malignancy like prostate cancer was found more frequently in the individuals with AS 19. Besides, the inflammatory reaction is an important factor for the formation of AS in which the inflammation and lipid deposition are two major mechanisms of the initial phase in the AS formation 20, 21. There are some inflammatory biomarkers that could increase under the condition of AS 22. The expression of both tumor growth factor beta and interleukin-6 would increase in the individuals with AS 23, and the nuclear factor-kappa B and bone morphogenic protein routes were significantly enhanced in the process of AS development 20. About the eyelid inflammatory diseases, the blepharitis resulted from the dysfunction and inflammation of meibomian gland 12, and the pro-inflammatory protein HMGB1 could elevated in the individuals diagnosed with blepharitis 24. On the other hand, the hordeolum also owns inflammatory nature in which the inflammatory response of the eyelid margin was observed in the individuals with hordeolum 25. Since both the AS and eyelid inflammatory diseases including blepharitis and hordeolum associate with the inflammatory reaction 12, 15, 25, the higher baseline inflammatory status in the individuals with AS may increase the rate of subsequent eyelid inflammatory diseases. This speculation was supported by the findings of this study at least to some extents.

The existence of AS correlated to higher incidence of blepharitis and hordeolum in this study. About the correlation between eyelid diseases and systemic diseases, the presence of coronary heart disease was significantly related to the higher incidence of subsequent blepharitis 26. In addition, the recurrent hordeolum was found in patient with nephrotic syndrome 27. Still, the association between AS and eyelid inflammatory diseases had not been fully surveyed. To our knowledge, our results may be a preliminary finding that the presence of AS significantly associated with the risk of succeeding blepharitis and hordeolum development. Moreover, the blepharitis and hordeolum episodes before the index date was excluded, this the time sequence between the AS and succeeding blepharitis and hordeolum may be established. Furthermore, we adjusted several confounders of blepharitis and hordeolum including the age, metabolic syndromes and the LDL/HDL concentration in the multivariable analysis. Consequently, the existence of AS may be the independent risk factor for the development of subsequent eyelid inflammatory diseases. In addition to the inflammation, the dysfunction of lipid also play a crucial role in the formation of blepharitis, hordeolum and AS 13, 16, 28, thus the higher serum lipid level in the AS may also trigger the development of succeeding blepharitis and hordeolum. About the cumulative incidences of eyelid diseases, both blepharitis and hordeolum showed a higher cumulative incidence in the AS group compared to the non-AS group. In the later years, the risk of eyelid inflammatory diseases gain 50% in the AS group compared to the similar values between groups within one year after the index date. These results may indicate the elevated risk of eyelid inflammatory diseases would be much higher in the individuals with prolonged AS than the non-AS population, which may influence the eyelid health to a higher degree.

Concerning the subgroup analysis, both the incidences of blepharitis and hordeolum were significantly higher in all the subgroups with AS except the Asian population and the individuals aged from 20-45 years. There was rare research to report such correlation. The young age was not a significant protective factor for the development of blepharitis in previous study 12, and the young adult also demonstrated average risk of developing hordeolum 25. On the other hand, the Asian population did not present with lower risk of blepharitis or hordeolum in previous articles 25, 29, and the rate of meibomian gland dysfunction was even higher in the Asian population. The possible explanation for the relative lower risk of eyelid inflammatory diseases in this study may due to the small case numbers in the Asian population and the young age group. Both subgroups account for less than 10 percent of cases in the whole study population, thus the small case numbers may contribute to the statistical bias. On the other hand, the risk of hordeolum in the African group with AS was also similar to the African group without AS. Because the total numbers of hordeolum episodes were relative fewer compared to the total numbers of blepharitis events (only about half), the low outcome numbers of hordeolum may also cause insignificant results in the population with relative low patient numbers. The AS correlated with higher incidence of eyelid inflammatory diseases in all the other subgroups compared to the non-AS population. These results may indicate the universal influence of AS on the development of eyelid inflammatory diseases.

Regarding the epidemiology, the AS is a common cardiovascular disorder in the world and the incidence of AS is near 3 percent in individuals older than 75 years in the North America are and the health of about 4.43 percent of US population was influenced by the AS 4, 30. Besides, the AS incidence in Chinese people aged older than 75 was more than 20 percent 31. The aortic valve replacement had been consumed in the majority of individuals for the AS in developed countries which is a large medical cost 32. For the surgical intervention, the operative mortality of aortic valve transplantation was more than 1 percent despite the survival time may elevate after early postoperative period 4, 11. The blepharitis is also a prevalent disease in which about 30 percent of individuals in ophthalmic department were diagnosed with blepharitis and many of them was suffered from dry eye 12. Also, the hordeolum may be one of the most common ocular diseases although the exact prevalence and incidence of hordeolum need further evaluation 25. Although the medical managements are adequate for most of hordeolum, the severe hordeolum may cause periorbital cellulitis which can influence the visual acuity 14. Since the AS, blepharitis and hordeolum influence a large portion of people and can contribute to huge socioeconomical burden, any association among them should be revealed.

There are several limitations in this study. Firstly, the TriNetX database is a claimed dataset which preserves the medical codes for the patients. Consequently, multiple important data including the site of AS, the severity of AS, the image result of AS, the surgical details of aortic valve replacement if arranged, the postoperative condition of individuals with AS if arranged, the recurrence of AS if existed, the appearance of eyelid inflammatory diseases, the meibomian gland condition of eyelid inflammatory diseases, the microorganism culture results of eyelid inflammatory diseases if arranged, the treatment outcomes of eyelid inflammatory diseases and the recurrence of eyelid inflammatory diseases if existed cannot be investigated. Secondly, there could be some individuals that had eyelid inflammatory diseases before the AS diagnosis but did not search medical assistance due to the light disease severity, thus the time sequence between AS and subsequent eyelid inflammatory diseases is not fully confirmed.

In conclusion, the existence of AS associates with higher incidence of succeeding eyelid inflammatory diseases including blepharitis and hordeolum after adjusting multiple confounders. Furthermore, the risk of developing eyelid inflammatory diseases in the AS population positively relates to the disease period of AS. Consequently, the routine ophthalmic referral may be suggested for the individuals with persistent AS. Further large-scale prospective study to evaluate the association between AS and therapeutic outcomes of eyelid inflammatory diseases is mandatory.

## Figures and Tables

**Table 1:** Baseline characteristics among aortic stenosis cohort and control cohort

Characteristics	AS cohort	Control cohort	SMD
N	431027	431027	
Age at Index	65.1±11.7	65.6±11.3	0.0385
Sex			
Female	181869 (42.2%)	179975 (41.8%)	0.0089
Male	236436 (54.9%)	236865 (55.0%)	0.0020
Race			
White	320968 (74.5%)	325208 (75.5%)	0.0227
Black or African American	46247 (10.7%)	43532 (10.1%)	0.0206
Asian	12340 (2.9%)	11174 (2.6%)	0.0166
Comorbidities			
Hypertensive diseases	287680 (66.7%)	291080 (67.5%)	0.0168
Dyslipidemia	214894 (49.9%)	216622 (50.3%)	0.0080
Ischemic heart diseases	174353 (40.5%)	173865 (40.3%)	0.0023
Diabetes mellitus	120962 (28.1%)	119623 (27.8%)	0.0069
Chronic kidney disease	106658 (24.7%)	102937 (23.9%)	0.0201
Peripheral vascular disease	98052 (22.7%)	94618 (22.0%)	0.0191
Chronic lower respiratory diseases	80255 (18.6%)	77223 (17.9%)	0.0182
Cerebrovascular diseases	68052 (15.8%)	65215 (15.1%)	0.0182
Diseases of liver	35786 (8.3%)	31642 (7.3%)	0.0358
Cerebrovascular disease	486 (0.1%)	805 (0.2%)	0.0191
Lab data			
eGFR	67.4±30.6	66.9±29.7	0.0176
HDL	46.4±18.5	46.4±18.6	0.0047
LDL	92.6±39.9	96.4±40.7	0.0941
Troponin	1.3±10.5	1.6±12.7	0.0249

AS: aortic stenosis, eGFR: estimated glomerular filtration rate, N: number, SMD: standard mean difference

**Table 2:** Main outcomes between the two groups

Study event	N	Cumulative probability				Hazard ratio (95% CI)	P
		1-year	3-years	5-years	10-years		
All events							
AS	6,165	0.44%	1.27%	2.07%	4.05%	1.270 (1.222-1.319)	<0.001*
Control	4,546	0.31%	0.98%	1.61%	3.38%	Reference	
Blepharitis							
AS	4,464	0.32%	0.91%	1.49%	2.99%	1.331 (1.271-1.393)	<0.001*
Control	3,139	0.21%	0.66%	1.11%	2.38%	Reference	
Hordeolum							
AS	2,184	0.15%	0.45%	0.73%	1.47%	1.183 (1.111-1.260)	<0.001*
Control	1,724	0.11%	0.37%	0.61%	1.31%	Reference	

aHR: adjusted hazard ratio, AS: aortic stenosis, CI: confidence interval, N: number * denotes significant difference between groups
